# Impact of cellular autophagy on viruses: Insights from hepatitis B virus and human retroviruses

**DOI:** 10.1186/1423-0127-19-92

**Published:** 2012-10-30

**Authors:** Sai-Wen Tang, Aurelie Ducroux, Kuan-Teh Jeang, Christine Neuveut

**Affiliations:** 1 Molecular Virology Section, Laboratory of Molecular Microbiology, National Institute of Allergy and Infectious Diseases, National Institutes of Health, Bethesda, Maryland, 20892-0460, USA; 2 Laboratory of Hepacivirus and Innate Immunity, Institut Pasteur, 75015, Paris, France

## Abstract

Autophagy is a protein degradative process important for normal cellular metabolism. It is apparently used also by cells to eliminate invading pathogens. Interestingly, many pathogens have learned to subvert the cell’s autophagic process. Here, we review the interactions between viruses and cells in regards to cellular autophagy. Using findings from hepatitis B virus and human retroviruses, HIV-1 and HTLV-1, we discuss mechanisms used by viruses to usurp cellular autophagy in ways that benefit viral replication.

## Review

### Background

The term “autophagy” means “self-eating” derived from Greek. It was first mentioned by Christian De Duve in 1963 [1], and has been used since to describe a bulk degradation process by lysosome-dependent mechanism. Autophagy functions to degrade protein aggregates, maintain the homeostasis of organelles, such as mitochondria, peroxisomes and ribosomes, and destroy intracellular pathogens [2]. The selectivity of autophagic degradation is thought to be achieved by recognizing post-modification such as ubiquitination [3] or acetylation on proteins [4,5]. Several autophagy receptors or adaptors, including SQSTM1/p62, NBR1 and HDAC6, have been identified, and they are considered to function by recognizing and recruiting ubiquitinated protein aggregates to be degraded through the autophagy pathway [6]. Until now, several types of autophagy-mediated degradation have been described. These include: 1) macroautophagy that is used to sequester cytoplasmic materials such as organelles and intracellular pathogens by *de novo* formation of double-layer membranes [7]: 2) microautophagy that is used to engulf a part of the cytoplasm by the invagination of lysosomal membrane into lysosome lumen [8]; 3) chaperone-mediated autophagy that is used to transport specific cytosolic proteins by chaperones to lysosomal degradation [9]. Macroautophagy will be discussed in this review and is herein referred to as autophagy.

### The autophagy machinery

**Figure 1:**
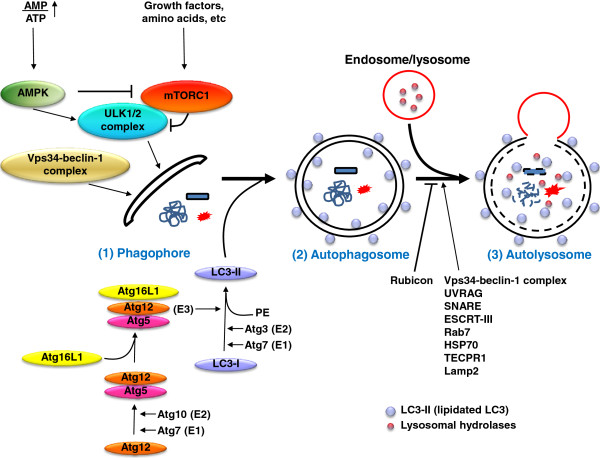
**A schematic summary of the autophagy machinery.** (1) The nucleation of phagophore membranes (pre-autophagosomal structures or isolation membrane): In nutrient rich condition, the mTORC1 kinase associates with the ULK1/2 complex to inhibit the initiation of autophagy. Under growth factor deprivation or nutrient starvation, energy sensor AMPK suppresses the activity of mTORC1 and activates the ULK1/2 complex which is essential for the induction of autophagy. The ULK1/2 complex likely recruits the Vps34-Beclin-1 complex to the site of autophagosome generation. (2) The formation of autophagosomes: Two ubiquitin-like proteins, Atg12 and LC3, are involved in the formation of enclosed double-membrane vesicles (autophagosomes) in order to sequester cytoplasmic material. Atg12 is conjugated with Atg5 by Atg7 (E1-like ubiquitin activating enzyme) and Atg10 (E2-like ubiquitin conjugating enzyme), which then form a complex with Atg16L1; this complex (E3-like ubiquitin ligase) works with Atg7 (E1) and Atg3 (E2) to conjugate LC3-I with phosphatidylethanolamine (PE), to create a form termed LC3-II, which is specifically located on autophagosome structures. (3) The maturation of autophagosomes: Autophagosomes are sequentially fused with endosomes and lysosomes to form autolysosomes. The lysosomal hydrolases degrade eventually the content of autophagosomes. To date, Vps34-Beclin-1 complex, UVRAG, SNARE, ESCRT-III, Rab7, HSP70 and TECPR1 have been identified to be involved in autophagosome-lysosome fusion. Rubicon protein may serve as a suppressor of autophagosome maturation by interacting with VPS34-Beclin-1 complex.

The autophagy machinery contains more than 30 autophagy-related (Atg) genes; most of which are highly conserved from yeast to mammal. When autophagy is induced by stressed conditions such as starvation, the first step is the nucleation of phagophore membranes (Figure 1), also called pre-autophagosomal structures [10] or isolation membrane, which likely originates from the endoplasmic reticulum, Golgi complex, mitochondria, endosomes and/or the plasma membrane [11]. In nutrient rich condition, the mammalian target of rapamycin (mTORC1) kinase is activated by class I PI3K and amino acids to inhibit the autophagy pathway by associating with and inactivating the ULK1/2 (Atg1 in yeast) complex (including ULK1/2, Atg13 and FIP200), which is essential for the induction of autophagy [12-14] (Figure 1). Under growth factor deprivation or nutrient starvation, the activity of mTORC1 is inhibited by energy sensor AMP activated protein kinase (AMPK). The ULK1/2 complex is also directly activated by AMPK-mediated phosphorylation, resulting in the translocation of ULK1/2 complex to the membrane of endoplasmic reticulum [14-17]. The ULK1/2 complex works mechanistically, likely through the recruitment of the Vps34 (class III PI3K)-Beclin-1 complex to the site of autophagosome generation to produce phosphatidylinositol-3-phosphate (PI3P), which is enriched on the inner surfaces of the phagophores, and to recruit PI3P binding proteins including WIPI-1 (Atg18 in yeast), small GTPase Rab5 and Atg14 for autophagy initiation [13,18-22] (Figure 1).

After phagophore membrane formation, the phagophores are elongated by two ubiquitin-like proteins, Atg12 and microtubule-associated protein 1 light chain 3 (LC3, Atg8 in yeast), to form enclosed double-membrane vesicles as known as autophagosomes in order to sequester a part of the cytoplasm (Figure 1). In this process, Atg12 is first activated by E1-like ubiquitin activating enzyme Atg7, transferred to E2-like ubiquitin conjugating enzyme Atg10, and then conjugated to a lysine residue (K130) of Atg5 [23]. The covalently linked Atg12-Atg5 and another membrane-bound factor, Atg16L1, further form a complex, which functions to expand the phagophore membrane; this complex dissociates from the membrane when autophagosomes are formed [24-26]. In a second step, full-length LC3 precursor is translated and immediately cleaved by the protease Atg4B to produce LC3-I (cytosolic form) with a free glycine residue [27,28]. Upon autophagy induction, LC3-I is conjugated to phosphatidylethanolamine [10] by the functions of E1-like ubiquitin activating enzyme Atg7 and another E2-like ubiquitin conjugating enzyme Atg3 to produce LC3-II [29-31]. The Atg12-Atg5-Atg16L1 complex has been reported to guide LC3-I to the phagophore membrane, and to function as E3-like ubiquitin ligase to promote the lipidation of LC3-I by PE [32,33]. LC3-II is specifically located on autophagosome structures, making it a commonly used specific marker for identifying autophagosomes [10]. LC3-II, which is located on inner membrane of autophagosomes, is eventually degraded after the fusion of the autophagosome with lysosome; however, the LC3-II protein on the outer membrane can be recycled and reused after delipidation by Atg4 [28].

A maturation step of autophagy is the sequential fusion of autophagosomes with endosomes and lysosomes to form autolysosomes; this fusion leads to the eventual degradation of the content of autophagosomes [34,35] (Figure 1). Recent reports suggested that SNARE [36], ESCRT-III [37], small GTPase Rab7 [38,39], and HSP70 [40,41] are involved in autophagosome maturation. Other relevant findings include that the UVRAG protein is able to interact with Vps34-Beclin-1 complex to activate GTPase Rab7 and autophagosome-lysosome fusion [42] and that the Rubicon protein suppresses the maturation of autophagosomes by interacting VPS34-Beclin-1 complex [43]. The latter observation indicates that the VPS34-Beclin-1 complex can also regulate autophagosome maturation depending on selective protein association. Additionally, the TECPR1 protein is thought to form a complex with Atg12-Atg5 and PI3P to enhance the fusion of autophagosomes with lysosomes [44]. At the same time, it should be noted that functional lysosomes are also needed for autophagosome maturation. Thus, a deficiency of Lamp2, which is an essential constituent of the lysosomal membrane, causes autophagosome accumulation and disrupts proper autophagy-mediated degradation [45]. Moreover, the disruption of lysosomal acidification by bafilomycin A (BFA, an inhibitor of the lysosomal vacuolar-ATPase) or chloroquine (a lysosomotrophic agent to increase pH in lysosomes) strongly impairs autophagosome-lysosome fusion [46,47]. The mechanism of how lysosomal acidification influences autophagosome-lysosome fusion, however, needs further exploration.

### Diseases associated with the mutation of autophagy-related genes

Genetic mutations of several autophagy-related genes have been linked to human diseases. For example, Beclin-1 has been suggested to suppress tumorigenesis and progression of breast cancer [48]. The monoallelic deletion of *Beclin-1* has been observed in 40-75% of human breast, ovarian, and prostate malignancies [49]. *UVRAG* is found to be monoallelically mutated in human colon cancer, and UVRAG has been suggested to act by inhibiting the proliferation and tumorigenic activity of human colon cancer cells [50,51]. By Genome-Wide Association Study (GWAS), IRGM1 (autophagy-stimulatory immunity-related GTPase) and Atg16L1 have been identified to be associated with the pathogenesis of chronic inflammatory bowel diseases, such as Crohn’s disease [48,52]. The somatic mutation of *LAMP-2* has been linked to Danon disease, which exhibits cardiac hypertrophy and the accumulation of autophagosomes and lysosomal glycogen in cardiac muscle cells causing clinical symptoms of cardiomyopathy, myopathy and mental retardation [53,54]. The deficiency of *LAMP-2* in mice also results in similar vacuolar cardioskeletal myopathy [53]. SQSTM1/p62 is an autophagy receptor, which recognizes and sends ubiquitinated substrates to be degraded by autophagy. Mutations in the ubiquitin associated (UBA) domain of SQSTM1/p62 have been reported to be associated with about 30% of Paget’s bone disease, which has disordered NF-κB-dependent osteoclast function and is characterized by focally increased and disorganized bone remodeling [55]. These collective examples raise the notion that perturbed activity of the autophagy pathway influences genomic instability and normal cellular metabolism [52].

### Autophagy and cancer

The link between autophagy and cancer development has been broadly established. Autophagy can clear toxic aggregates and damaged mitochondria which may produce reactive oxygen species (ROS) that cause DNA damage [56,57], and autophagy has attributed roles in chromosome instability, including aneuploidy and gene amplification [58-60]. Moreover, a deficiency of autophagy results in failed degradation of SQSTM/p62, which plays a role in activation of NF-κB and inflammation-mediated tumorigenesis [56,61,62]. Thus, conceptually, autophagy serves to reduce environmental insults and neutralize events that favor cellular transformation. Indeed, in cellular transformation, it has been commonly regarded that apoptosis provides a protective mechanism in inciting the death of aberrantly transformed cells. In that context, it is increasingly recognized that apoptotic cell death of abnormal cells can be complemented by apoptosis-independent autophagy-dependent cell death [61,63], especially in the elimination of transformed cells.

In a related aspect, the function of autophagy as a provider of nutrient and energy also contributes to tumor survival, especially under metabolically stressful condition such as nutrient starvation and hypoxia [64]. This concept is supported by the clinical observation that biallelic loss of *Beclin-1* has not been seen in cancer patients [49,65], and by *in vitro* experiments showing that autophagy deficiency achieved by small interfering RNA targeting Beclin-1 or Atg5 reduces cellular proliferation and increases the death of cancer cells [61,66,67]. Additionally, activation of autophagy is observed within cancer cells treated with chemotherapy or radiotherapy. Thus although these cancer therapies are designed to kill most cancer cells, it is a concern that by triggering increased autophagy they incite a reactive response that helps the residual cancer cells survive and resist extreme stress [64]. It is thus reasonable to consider a cancer treatment approach that combines traditional anti-cancer chemotherapy with autophagy inhibitors such as hydroxychloroquine. Several clinical trials are underway examining the effect of autophagy inhibitors on increasing the sensitivity of cancers to chemotherapy [68,69].

### Autophagy and pathogen clearance

Autophagy also functions as a cellular defense to remove invading pathogens, in a process termed xenophagy; and autophagy can serve to deliver antigen fragments of pathogens for MHC class II presentation to activate the adaptive immune system [70]. Many types of bacteria have been reported to be targeted by autophagic degradation. For example, *Mycobacterium tuberculosis* can be targeted by autophagy, and its clearance is enhanced by cellular starvation and exposure to lipopolysaccharides. The clearance of *Toxoplasma gondii* can be decreased by treatment with Bafilomycin A (an autophagy inhibitor) or Beclin-1 siRNA [71]. Ubiquitination of proteins is likely a crucial step for the clearance of invading bacteria. NDP52 (nuclear dot protein 52kDa) functions as an autophagy receptor that recognizes ubiquitinated *Salmonella enterica* and captures it into autophagosomes by interacting with LC3 [72]. Recently, Watson *et al*. have observed that the cellular STING-dependent pathway recognizes extracellular bacterial DNA, triggering the intracellular ubiquitination of bacterial proteins, and that SQSTM/p62, NDP52 and the DNA-responsive kinase TBK1 are used for autophagic degradation of bacteria [73]. However, it should be noted that many bacteria have evolved countermeasures to combat the cell’s autophagic defense. For example, *Legionella pneumophila* and *Brucella abortus* do induce cellular autophagy, but can thwart the maturation step of autophagy in order to facilitate pathogenic replication [70]. Similarly, *Mycobacterium tuberculosis* can interfere with autophagosome-lysosome fusion through its ESAT-6 Secretion System-1 (ESX-1) [74].

### Autophagy and viruses

Interestingly, most viruses, with a few exception such as vesicular stomatitis virus (VSV), appear to have evolved mechanisms to evade cellular clearance by autophagy [75]. Many viruses have developed counteracting mechanisms to escape autophagic degradation [66,76]. For instance, several herpesviruses, including herpes simplex virus type 1 (HSV-1), bovine herpesvirus type 1 (BHV-1), human cytomegalovirus (HCMV), Kaposi’s sarcoma-associated herpesvirus (KSHV), herpesvirus saimiri (HVS) and molluscum contagiosum virus (MCV), can capably suppress autophagy. For HSV-1, the viral protein ICP34.5 interacts with Beclin-1 to inhibit autophagy induction [77,78].

Some DNA viruses including Epstein-Barr virus (EBV), varicella-zoster virus (VZV), adenovirus, human papillomavirus 16 (HPV16), simian virus 40 (SV40), human parvovirus B19 (HPV-B19) and hepatitis B virus (HBV), activate portions of the autophagy pathway and employ this process to enhance viral replication. Thus, autophagy-induced cell death assists the final step of the adenovirus life cycle to release virus particles [79]. Many RNA viruses, including VSV, coxsackievirus B4 (CVB4), coxsackievirus B3 (CVB3), poliovirus, dengue virus-2 (DENV2), dengue virus-3 (DENV3), rotavirus, hepatitis C virus (HCV), influenza virus A, have been observed to induce autophagy, but inhibit autophagosome-lysosome fusion. [80]. For poliovirus and HCV, autophagy induction seems to provide cell membranes for RNA replication [81-84]. For influenza A virus, the viral M2 protein inhibits autophagosome-lysosome fusion, possibly inhibiting MHC antigen presentation of influenza A virus proteins to reduce host immune response [85]. Below, we will discuss in greater depth lessons on autophagy learned from hepatitis B virus and human retroviruses.

### Lessons learned from HBV

The human hepatitis B virus (HBV) is the prototype member of a family of small, enveloped DNA viruses called Hepadnaviridae that infect a restricted number of mammals and birds. Despite the existence of effective vaccines, HBV remains one of the most significant human pathogens with an estimated 2 billion people infected worldwide, of whom 350 million are chronic HBV carriers. Chronic hepatitis B is a major risk factor for severe liver diseases including liver cirrhosis and hepatocellular carcinoma (HCC). HCC is the fifth most common cancer, and the third leading cause of cancer death in the world [86].

Although chronic HBV infection has been epidemiologically linked to the development of HCC for more than 40 years, the mechanisms by which HBV infection results in HCC are still unclear. The hepatitis B virus X protein (HBx) has generally been viewed as an oncoprotein in viral carcinogenesis. In order to favor virus replication, HBx subverts cellular activities such as signal transduction, transcription, autophagy, and proliferation. In doing so, HBx apparently induces the accumulation of cellular dysfunctions and damage, ultimately leading, in the case of viral persistence, to cancer development.

**Figure 2:**
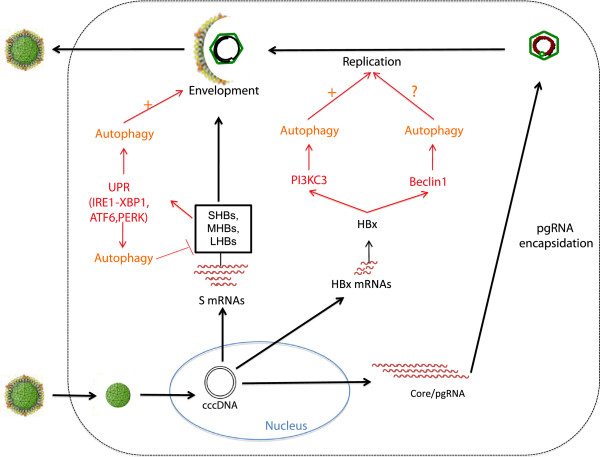
**Autophagy is induced by HBV expression and enhances HBV replication.** HBV increases autophagy to favor its own replication. The exact steps impacted by autophagy are still unclear, but it seems that autophagy can either enhance HBV DNA replication or favor HBV envelopment. To date, different non-exclusive mechanisms for autophagy induction have been proposed. The regulatory protein HBx could directly activate autophagy through the induction of PI3KC3 activity or the up-regulation of beclin1 expression. Finally the small envelope protein (SHBs) has been shown to induce autophagy via the establishment of ER stress that triggers the unfolded protein response (UPR) and autophagy. Interestingly, a study has reported that induction of UPR triggers autophagy-dependent degradation of three HBV envelope proteins. This mechanism seems to be in contradiction with the previous finding. It will be interesting to determine if this autophagy-dependent mechanism regulates the overall production of viral particles following autophagy induction.

HBV enhances and uses autophagy for its replication. However, the mechanisms responsible for autophagy induction as well as the step of HBV replication affected by autophagy are still controversial [87,88]. Upon viral infection, autophagy can be triggered by direct mechanisms like the recognition of viral element that promote autophagy protein expression, or by indirect mechanisms like virus triggered cellular stress. For example, during infection a large amount of viral proteins are synthesized and unfolded, and the misfolded proteins can activate ER stress response. HBV can use direct and indirect mechanisms to induce autophagy (Figure 2).

Different groups have shown that HBV expression is correlated with autophagy induction [87-89]. Two of these publications observed that while HBV expression induces the formation of early phagosomes, the rate of autophagic protein degradation is seemingly not increased [87,88,90]. These results argue that HBV acts on the early step of phagosome formation. As noted above, some viruses induce early steps in the autophagy process, but delay phagosome maturation, in order to promote viral replication [91,92]. Further work will be needed to determine carefully whether and how HBV blocks the formation of late phagosomes. Interestingly, Tang and collaborators have observed autophagy induction by HBV only under starvation condition. The discrepancy between this work and other studies could stem from the different cell lines used in these studies. Alternatively the models used: transfected HBV genome versus integrated HBV genome could lead to different levels of viral protein expression, and increasing the level of HBV protein can induce ER stress [93] thereby activating autophagy [94]. For future clarity, it will be necessary to assess autophagy in the context of authentic HBV infection.

The exact steps of HBV replication that are regulated by autophagy remain to be identified. In extant publications the role of autophagy on the early steps of HBV infection is not addressed; however, current data do support that autophagy impacts late steps of HBV replication, increasing HBV production. Indeed, using either an inhibitor of PI3KC3 or via the silencing of enzymes essential for the formation of autophagosomes, Sir *et al*. showed that inhibition of autophagy had a marginal effect on HBV transcription and HBV RNA packaging, but suppressed HBV DNA synthesis, suggesting an enhancement of HBV DNA replication by autophagy [88]. They further confirmed the role of autophagy in the production of HBV virions *in vivo* using HBV transgenic mice with liver-specific knockout of Atg5. They demonstrated in this model that the formation of autophagosome is essential for HBV DNA synthesis in the cytoplasm [90]. Again, in this study the HBV DNA is integrated into the mouse genome, preventing direct extrapolation of the findings to *in vivo* HBV infection. Moreover, these studies are somewhat in contradiction with the study of Li and collaborators who reported that the autophagy machinery is needed for efficient envelopment of the nucleocapsids at the ER membrane and has only a slight effect on HBV DNA replication [87]. It is unclear the reasons for the differences; in both studies, the HBV genome is transfected into hepatoma cells, albeit using different techniques. However, it may be that slight differences in the level of viral protein expression and the cell lines employed could account for the discrepancies between the two studies.

If the impact of autophagy on HBV replication remains a matter of discussion, the mechanisms leading to the induction of autophagy by HBV remain also unclear (Figure 2). It was first suggested that the viral regulatory protein HBx was directly involved in starvation-induced autophagy via the up–regulation of Beclin-1 expression [89]. In that report, the authors showed that HBx, a known weak transcriptional activator, transactivates the Beclin-1 promoter in hepatic and hepatoma cell lines. They next demonstrated that silencing of Beclin-1 expression by siRNA blocked the induction of autophagy by HBx, suggesting that HBx acts via the transcriptional activation of Beclin-1. These authors, however, did not include control experiments with a transactivation-deficient HBx mutant and their siRNA knock down results do not formally address increased Beclin-1 transcription by HBx, rather the findings solely indicate that Beclin-1 is essential for induction of autophagy by HBx. This is an important point because in another publication, Sir *et al*. did not observe induction of Beclin-1 expression by either HBV or HBx. Rather, those investigators observed that HBx interacted with PI3KC3 and enhanced the latter’s activity [88].

HBV can also induce autophagy indirectly via the induction of cellular stress [94-96] (Figure 2). In searching for the mechanism leading to autophagy upon HBV expression, Li and collaborators found that the expression of HBV small surface protein (SHBs) induced ER stress and subsequently the activation of three signaling pathways PERK, ATF6 and IRE1. They further demonstrated that the blockade of any of these three UPR (unfolded protein response) signaling pathways blocked autophagy induction. Their study supports the idea that induction of ER stress by SHBs is the inducer of autophagy [87]. Moreover, the authors observed an interaction between SHBs proteins and the autophagosome marker LC3, suggesting that this interaction could be involved in the enveloping process of HBV virions (Figure 2). How autophagy enhances viral envelope acquisition needs further investigation. One should note that another group has reported findings in contradiction with the notion that SHBs proteins increase autophagy without enhancing the rate of protein degradation or that autophagy favors virus replication. Indeed, Lazar and collaborators showed that HBV activates UPR through the increase of EDEM1 expression, which negatively controls viral particle production [97]. They demonstrated that EDEM1 expression leads to the degradation of HBV envelope proteins L, M and S by autophagy. However, Lazar and coworkers studied the role of EDEM1 on viral surface protein stability in HEK293T cells that over-expressed viral envelope proteins. Whether envelope proteins are degraded in the setting of authentic HBV infection and replication was not addressed.

### Lessons learned from human retroviruses

Human immunodeficiency virus-1 (HIV-1) is the causative agent for acquired immunodeficiency syndrome (AIDS) [98,99]; the virus infects over 30 million individuals worldwide and causes approximately 3 million deaths each year. HIV-1 infects and replicates in CD4^+^ T cells and macrophages [100,101]. After entry into cells, HIV-1 replication is challenged by cellular autophagic degradation [102] and/or by host cell restriction factors [103-105], such as APOBEC3G [106,107], BST-2/Tetherin [108-110], TRIM5α [111,112], SAMHD1 [113-116], and microRNAs [117,118]. However, HIV-1 has evolved means to counter these defense mechanisms to overcome these cellular restrictions. For example, HIV-1 uses viral accessory protein Vif to promote the degradation or exclude the virion incorporation of APOBEC3G [106,119]; the Vpu protein to counter the effect of BST-2/Tetherin [109,120,121], and the Tat protein to modulate cellular miRNA activity [122,123].

**Figure 3:**
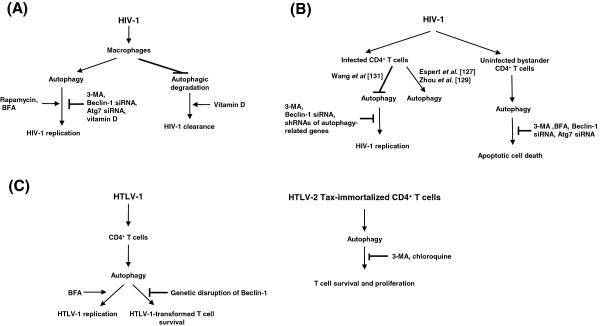
**Human retroviruses subvert autophagy.** (**A**) HIV-1 infection of macrophages induces autophagy to enhance viral replication. Inhibition of autophagy by 3-MA, Beclin-1 siRNA, Atg7 siRNA and vitamin D has been demonstrated to reduce HIV-1 replication [125,126,128]. Additionally, HIV-1 Nef protein inhibits autophagic clearance of HIV-1 by blocking the autophagosome-lysosome fusion; treatment of vitamin D overcomes this blocking to enhance HIV-1 clearance [128]. (**B**) The autophagic effect of HIV-1 infection in CD4^+^ T cells is still controversial. Espert *et al*. and Zhou *et al.* observed that HIV-1-infected CD4^+^ T cells exhibits reduced autophagy [127,129]. However, Wang *et al.* reported that HIV-1 infection of CD4^+^ T cells activates autophagy and that treatment of autophagy inhibitor 3-MA and Beclin-1 siRNA suppresses HIV-1 replication [131]. Eekels *et al.* performed shRNA-mediated stable knockdown of autophagy-related genes (such as Atg5 and Atg16) and showed an inhibitory effect on HIV-1 production [132]. In uninfected bystander CD4^+^ T cells, circulating HIV-1 *env* protein activates autophagy to cause apoptotic cell death, which can be inhibited by treatment of 3-MA and BFA or Beclin-1 siRNA and Atg7 siRNA [127,133,134]. (**C**) HTLV-1-infected T cells exhibit an increase of autophagy for its survival and viral replication [147] (Tang *et al*., submitted). Genetic disruption of Beclin-1 reduces the viability of HTLV-1-transformed T cells [147], and BFA treatment enhances HTLV-1 replication (Tang *et al*., submitted). HTLV-2 Tax-immortalized CD4^+^ T cells show increased autophagy, which is essential for its survival. Autophagy inhibitors 3-MA and chloroquine suppress the proliferation and induce the apoptosis of HTLV-2 Tax-immortalized T cells [148]

Regarding autophagy, HIV-1 apparently subverts this cellular defense process in a manner to benefit viral replication. In macrophages, the viral accessory protein Nef [124,125], by interacting with Beclin-1, has been found to block the maturation step of autophagy and thus acts to prevent the destruction of HIV-1 [126] (Figure 3A). Treatment of BFA, an inhibitor of autophagosome-lysosome fusion, enhances accordingly HIV-1 production [127]. Additionally, immunity-associated GTPase family M (IRGM), which interacts with Atg5 and Atg10, has been reported to be another target of Nef for the accumulation of autophagosomes and HIV-1 production [80]. It has been observed that a Nef-deficient HIV-1 cannot overcome autophagic degradation and replicates less efficiently [126]. Overall, the current findings are that the early steps of autophagy contributes to HIV-1 replication (Figure 3A), and consistent with this notion, HIV-1 Gag is seen colocalized with LC3-enriched autophagosomes; and treatment of cells with the autophagy inhibitor 3-methyladenine (3-MA) and siRNA-mediated knockdown of Beclin-1 or Atg7 significantly reduces the yield of HIV-1, while the autophagy inducer rapamycin enhances virus production [126]. A recent study also found that vitamin D treatment can inhibit HIV-1 replication through initiating and promoting the maturation of autophagy, and that treatment of cells with BFA and knockdown of Beclin-1 and Atg5 counter the inhibitory effect of vitamin D [128]. These results suggest approaches that increase autophagosome-lysosome fusion could potentially be useful anti-HIV-1 therapeutic strategies.

HIV-1 infection of CD4^+^ T cells is not identical to its infection of macrophages. Unlike reports from macrophages, HIV-1 infection in MOLT-4 T lymphoblast cell line and CD4^+^ T cells has been suggested by one research group to inhibit autophagy as measured by reduced LC3-II or Beclin-1 levels [127,129] (Figure 3B); this indicates that in T-cells the process of autophagy may be a net negative for HIV-1 replication [130]. However, this notion is somewhat unsettled because another group has reported that autophagy is induced by HIV-1 infection of CD4^+^ T cells, as shown by increased levels of Beclin-1 and LC3 [131] (Figure 3B). Moreover, they found that the levels of ULK1, Atg4D, Atg5 and Atg12 conjugates were also increased by HIV-1 or HIV-2 infection, and that autophagy inhibitor 3-MA and Beclin-1 siRNA were able to inhibit HIV-1 replication in Jurkat T cells [131]. Elsewhere, the stable knockdown of autophagy-related genes, such as Atg5 and Atg16 also was seen to inhibit HIV-1 production in SupT1 cells [132]. These results suggest that activation of autophagy is a net positive for HIV-1 in T cells; indeed, the relationship between HIV-1 and autophagy in T-cells remains incompletely understood and requires further investigation.

Further complicating the picture is a postulated role of HIV-1 on the autophagy status of uninfected bystander CD4^+^ T cells. Through interacting with CXCR4 or CCR5, soluble circulating HIV-1 *env* protein induces autophagy to trigger apoptosis in uninfected CD4^+^ T cells (Figure 3B), accounting in part for the clinical depletion of CD4^+^ T cells [127,133,134]. The apoptotic cell death induced by *env* protein can be fully inhibited by treating cells with 3-MA and BFA, or using siRNAs to knock down Beclin-1 and Atg7, indicating a link between autophagy and apoptosis through autophagy-related proteins [130,133].

Another human retrovirus is the Human T-cell Leukemia Virus type 1 (HTLV-1), which was identified a few years prior to HIV-1, and is the etiological agent for a human lymphoproliferative malignancy, adult T-cell leukemia (ATL), and chronic inflammatory diseases, including HTLV-1-associated myelopathy (HAM)/tropical spastic paraparesis (TSP) [135-138]. HTLV-1 infects approximately 10 to 20 million individuals worldwide [139]. The virus infects CD4^+^ T cells, CD8^+^ T cells, B cells, macrophages and fibroblasts; this diversity of infection occurs possibly because of the ubiquitous distribution of its hypothesized receptors (glucose transporter 1, heparan sulfate and proteoglycans and neuropilin-1) [140,141]. Empirically, HTLV-1 primarily targets CD4^+^ T cells, resulting in persistent NF-κB activation by the viral regulatory protein Tax, leading to the clonal expansion of CD4^+^ T cells [142-146].

A recent report showed that HTLV-1-infected T cells exhibit increased autophagy and that the genetic disruption of Beclin-1 decreased the viability of HTLV-1-transformed T cells [147] (Figure 3C). HTLV-1 Tax was found to interact with the Vps34-Beclin1 complex in IKKβ –dependent fashion [147]. Additionally, HTLV-1 infection and Tax expression have been found to induce autophagy; and in this setting, blocking the autophagosome-lysosome fusion was shown to benefit virus replication (Tang *et al*., submitted) (Figure 3C). Mechanistically, the ability of HTLV-1 Tax to activate NF-κB pathway correlates with its induction of autophagosome accumulation (Tang *et al*., submitted).

Separately, it has been reported that CD4^+^ T cells immortalized by HTLV-2 Tax protein have increased LC3-II compared to Jurkat T cells, and that autophagy inhibitors (3-MA and chloroquine) inhibit the proliferation and induce the apoptosis of HTLV-2 Tax-immortalized T cells [148] (Figure 3C). HTLV-2 Tax was shown to interact with Vps34, IKKβ and Beclin-1, and shRNA-mediated knockdown of IKKβ or Beclin-1 expression reduced HTLV-2 Tax-induced accumulation of LC3-II, providing a possible mechanism for how HTLV-2 Tax activates autophagy [148]. Going forward, a comparison of similar/different mechanism(s) shared by HTLV-1 and −2 Tax proteins in autophagy induction would be informative.

A recent study suggested that the degradation of IKK (inhibitor of kappa B kinase) induced by geldanamycin inhibition of Hsp90 (heat shock protein 90) is through the autophagy, not the proteasome, pathway. In Atg5-deficienct cells with impaired autophagy, IKK degradation induced by geldanamycin treatment is attenuated, indicating that in this setting, autophagy plays a key role [149]. Additionally, treatment with autophagy inhibitors increased the survival of ATL cells when their Hsp90 protein is inhibited by geldanamycin treatment [150,151]. These results implicate autophagy as playing a physiological role in the death of ATL cells.

## Conclusions

Autophagy is a highly conserved process used to regulate cellular metabolism and to protect cells against invading pathogens. Accumulating findings have, however, suggested that many pathogens have evolved countermeasures to overcome the cell’s autophagic defense. Currently, a few bacteria strains and many virus types have adopted means to evade and usurp the autophagic process. Indeed, the ability to block autophagosome-lysosome fusion seems to be a common mechanism used by many viruses to induce autophagosome membrane generation; these viruses have evolved mechanisms to interrupt autophagosome destruction by preventing its fusion with lysosome. A number of viruses have adapted to utilize autophagosome membranes for the efficient replication of their viral genomes. As we increasingly understand virus-cell interaction, it appears that pharmaceutical agents that enhance autophagosome-lysosome fusion might be useful clinical tools. Recently, Campbell *et al*. found a promoting effect of vitamin D on autophagosome-lysosome fusion [128], raising the possible use of vitamin D in the clinical treatment of autophagy-related diseases, such as virus infection, cancers, and protein aggregate-related neurodegenerative diseases. The discovery of additional useful autophagy inducing and inhibiting molecules promises to be an exciting and fruitful area for future research.

## Competing interests

The authors have no competing financial interests.

## Authors' contributions

SW and AD wrote the manuscript; KTJ and CN critically edited the manuscript. All authors read and approved the final manuscript.
